# Epstein-Barr virus-specific intrathecal oligoclonal IgG production in relapsing-remitting multiple sclerosis is limited to a subset of patients and is composed of low-affinity antibodies

**DOI:** 10.1186/s12974-014-0188-1

**Published:** 2014-11-13

**Authors:** Massimiliano Castellazzi, Carlo Contini, Carmine Tamborino, Francesca Fasolo, Gloria Roversi, Silva Seraceni, Roberta Rizzo, Eleonora Baldi, Maria Rosaria Tola, Tiziana Bellini, Enrico Granieri, Enrico Fainardi

**Affiliations:** Department of Biomedical and Specialist Surgical Sciences, University of Ferrara, Via Aldo Moro 8, I-44124 Cona Ferrara, Italy; Department of Medical Sciences, University of Ferrara, Via Aldo Moro 8, I-44124 Cona Ferrara, Italy; Department of Neurosciences and Rehabilitation, Azienda Ospedaliero-Universitaria, Via Aldo Moro 8, I-44124 Cona Ferrara, Italy

## Abstract

**Background:**

The purpose of this study was to investigate intrathecal production and affinity distributions of Epstein-Barr virus (EBV)-specific antibodies in multiple sclerosis (MS) and controls.

**Methods:**

Cerebrospinal fluid (CSF) and serum concentrations, quantitative intrathecal synthesis, oligoclonal bands (OCB) patterns and affinity distributions of anti-Epstein Barr virus (EBV) antibodies were evaluated in 100 relapsing-remitting MS (RRMS) patients and 200 age- and sex-matched controls with other inflammatory neurological disorders (OIND) and other noninflammatory neurological disorders (NIND).

**Results:**

Levels of anti-EBNA-1 and anti-viral capsid antigen (VCA) IgG were different in both the CSF (*P* <0.0001 and *P* <0.01, respectively) and serum (*P* <0.001 and *P* <0.05, respectively) among the RRMS, OIND and NIND. An intrathecal synthesis of anti-EBNA-1 IgG and anti-VCA IgG, as indicated by the antibody index, was underrepresented in the RRMS, OIND and NIND (range 1 to 7%). EBV-specific OCB were detected in 24% of the RRMS patients and absent in the controls. High-affinity antibodies were more elevated in the RRMS and in the OIND than in the NIND for CSF anti-EBNA-1 IgG (*P* <0.0001) and anti-VCA IgG (*P* <0.0001). After treatment with increasing concentrations of sodium thiocyanate, the EBV-specific IgG OCB had low affinity in all 24 RRMS patients analyzed.

**Conclusions:**

Our findings do not support the potential role of an EBV persistent brain chronic infection in MS and suggest that an EBV-specific intrathecal oligoclonal IgG production can occur in a subset of MS patients as part of humoral polyreactivity driven by chronic brain inflammation.

## Background

Multiple Sclerosis (MS) is a chronic inflammatory demyelinating and neurodegenerative disease of the central nervous system (CNS) of supposed autoimmune origin, which is currently believed to be mediated by a combined attack directed by both T and B cells [1]. Although disease etiology remains largely unknown, epidemiological observations suggest the potential implication of an infectious organism as a causative agent of MS [2]. In this setting, an ideal candidate is represented by Epstein-Barr virus (EBV), a human γ-herpesvirus with a widespread distribution in the human population, which can infect and activate B-lymphocytes and persists latently for life [3]. Seroepidemiological studies have shown that there could be a strong association between MS and EBV. A past infectious mononucleosis (IM) was found to be more frequent, and the seroprevalence of anti-Epstein Barr nuclear antigen 1 (EBNA-1) and anti-viral capsid antigen (VCA) IgG was higher in MS patients than in controls [4–6]. High serum levels of anti-EBNA-1 IgG increased the risk of developing MS [7], correlated with disease activity [8] and predicted the conversion from clinical isolated syndrome (CIS) to definite MS [9]. Elevated serum concentrations of anti-VCA IgG were related to gray matter atrophy [10]. The role of EBV in MS pathogenesis was in part supported by the experimental demonstration that EBV proteins and myelin-basic protein epitopes share structural similarity [11]. However, conflicting results have been obtained in cellular, molecular and neuropathological studies since, in MS patients, blood EBV-specific CD8+ T cell response was found increased, decreased or absent; cerebrospinal fluid (CSF) and blood EBV DNA load was high or not measurable; and the detection of EBV-infected B cells in brain lesions was inconsistent [3,5,7,12]. Controversial findings were also reported in quantitative and qualitative analysis of intrathecal synthesis of anti-EBV IgG in MS. An antibody index (AI) suggestive of intrathecally produced anti-EBV IgG was more represented [13] or equivalent [14–19] in MS patients compared to controls, whereas the detection of CSF-restricted EBV-specific IgG oligoclonal bands (OCB) in MS patients was highly variable, ranging from 0% to 44% [16,20–24]. Nevertheless, none of the previous studies investigated the affinity distributions of intrathecally released anti-EBV antibodies. Therefore, the actual relevance of EBV in MS still remains to be elucidated. In this regard, it is particularly crucial to determine the exact nature of EBV-specific intrathecal humoral immune response since the key feature of chronic CNS infections is the presence of targeted intrathecaly produced high-affinity oligoclonal antibodies, of which only 20% are specific to the causative agent [2]. To address the question of whether an EBV persistent brain infection exists in MS, in this study we sought to verify the frequency of EBV-specific oligoclonal IgG restricted to CSF and their affinity distributions in a large number of MS patients and controls.

## Methods

### Study design

This study included 100 consecutive patients with relapsing-remitting definite MS (RRMS) according to the currently accepted criteria [25] (Table 1) followed by the MS Center of Ferrara (Italy) during the period from June 2004 to December 2008. MS relapse was defined as the onset of new or recurrent symptoms or signs or the worsening of already present neurological abnormalities persisting for at least 24 h in the absence of fever and preceded by at least 1 month of stable or improved neurological state [26]. Evidence of a relapse at admission was considered as clinical disease activity. At the time of sample collection a) the disease severity was scored using Kurtzke’s Expanded Disability Status Scale (EDSS) [27]; b) the disease duration was scored and expressed in months; c) the presence of relapse was recorded as clinical activity; and d) the lesions showing Gd-enhancement on T1-weighted scans were defined as MRI activity. At the moment of admission, any MS patients with fever or other symptoms of acute infection or treated with immunosuppressive (for example, azathioprine or methylprednisolone) or immunomodulatory (for example, interferon-beta or glatiramer acetate) drugs in the 6 months before entering the study were excluded. One hundred patients with other inflammatory neurological disorders (OIND) and 100 subjects with other non-inflammatory neurological disorders (NIND) were selected as neurological controls (Table 2); they were age and sex matched to RRMS according to previously proposed criteria [12]. None of the OIND and NIND patients were undergoing treatment with immunosuppressant drugs, including steroids, at the time of sampling.

**Table 1:** **Demographic, clinical, radiological and cerebrospinal fluid (CSF) characteristics in 100 patients with relapsing-remitting multiple sclerosis (RRMS)**

**Sex: F/M**	**70/30**
Age, years: mean ± SD	37.3 ± 10.6
Disease duration, months: mean ± SD	30.5 ± 46.6
Disease severity, EDSS: mean ± SD	2.0 ± 1.3
CA MS: n/total (%)	77/100 (77%)
CS MS: n/total (%)	23/100 (23%)
Gd+ MS: n/total (%)	37/100 (37%)
Gd- MS: n/total (%)	63/100 (63%)
Blood-CSF-barrier dysfunction, QAlb: positive/total (%)	18/100 (18%)
Intrathecal IgG synthesis, IgG Index: positive/total (%)	71/100 (71%)
CSF-restricted oligoclonal IgG bands: positive/total (%)	84/100 (84%)

CA, clinically active (presence of relapse at entry); CS, clinically stable (absence of relapse at entry); EDSS, expanded disability status scale; Gd+, appearance of gadolium enhancing lesions on magnetic resonance imaging (MRI); Gd-, no evidence of gadolium enhancing on MRI; QAlb, CSF/serum albumin quotient.

**Table 2:** **Demographic and clinical features in other inflammatory neurological disorders (OIND) and noninflammatory neurological disorders (NIND)**

**Patients**	**n**	**Sex**		**Age (years)**	**Type of disease**
		**F**	**M**		
OIND	100	70	30	37.5 ± 10.7	
	29				Chronic inflammatory demyelinating polyneuropathy
	18				Viral encephalomyelitis
	18				Acute inflammatory demyelinating polyneuropathy
	11				Bacterial meningitis
	10				Optic neuritis
	6				HIV encephalopathy
	3				Neurolupus
	3				NeuroSjogren
	3				NeuroBechet
NIND	100	70	30	38.1 ± 11.1	
	14				Transient ischemic attack
	13				Epilepsy
	12				Headache
	10				Cervical spondylosis
	10				Hereditary ataxia
	9				Vascular dementia
	9				Migraine
	8				Amyotrophic lateral sclerosis
	7				Compression neuropathy
	4				Paresthesias
	4				Alzheimer disease

NIND, noninflammatory neurological diseases; OIND, other inflammatory neurological diseases.

### Cerebrospinal fluid and serum sampling

Paired CSF and serum samples prospectively collected from RRMS, OIND and NIND patients were obtained for purposes of diagnosis and measured under exactly the same conditions. CSF samples were obtained by atraumatic lumbar puncture performed for the purpose of diagnosis in the absence of contraindications. All CSF and serum analysis were performed within 2 weeks of the onset of clinical symptoms in relapsing MS patients, and at least 2 months after the end of clinical exacerbation in clinically stable MS patients. Both cell-free CSF and serum samples were obtained after centrifugation at 3,000 rpm at 20°C for 15 minutes. Supernatants were collected, under sterile conditions, in aliquots of 500 μl, coded, frozen and stored at -80°C until assay. CSF and serum IgG and albumin levels were measured by immunochemical nephelometry with the Beckman Immage 800 system (Beckman Instruments, Fullerton, CA, USA). In all patients, blood-CSF-barrier (B-CSF-B) dysfunction was determined by the CSF/serum albumin quotient (QAlb) [28]. QAlb values >6.5 × 10^3^, >8.0 × 10^3^ and >8 to 9 × 10^3^ were considered indicative of B-CSF-B dysfunction for patients aged 16 to 40 years, aged 40 to 60 years and over 60 years, respectively. Total IgG OCB were routinely determined in paired CSF and serum samples by isoelectric focusing (IEF) using a commercially available kit (IgG IEF, Helena Laboratories, Gateshead, Tyne and Wear, UK). Results were interpreted by two independent observers (MC and EF). Five different profiles were identified according to the currently accepted guidelines [29] (normal = normal CSF; local synthesis = CSF restricted OCB; mixed = CSF restricted OCB with additional, identical bands in CSF and serum; mirror = identical bands in CSF and serum; and monoclonal = monoclonal bands in CSF and serum). Only the detection of local synthesis and mixed patterns was considered suggestive of intrathecal oligoclonal IgG synthesis. Conversely, a ‘mirror’ pattern reflected a systemic oligoclonal IgG response. The approvals of the Committee for Medical Ethics in Research of the University of Ferrara has been obtained for experiments involving human subjects. Written informed consent was obtained from all subjects participating in the study.

### Magnetic resonance imaging analysis

All MS patients underwent brain magnetic resonance imaging (MRI) scans at entry on a 1-Tesla MRI unit (GE Signa Horizon, General Electric Medical Systems, Milwaukee, WI, USA). Routinely used T1- weighted axial spin echo images were obtained approximately 10 min after intravenous injection of 0.1 mmol/kg of Gd-DTPA in each patient. Lesions showing Gd-enhancement on T1-weighted scans were defined as active. All brain MRI scans were evaluated by one investigator blinded to clinical and sample data.

### Serum and cerebrospinal fluid levels and intrathecal synthesis of anti-Epstein-Barr virus antibodies

CSF and serum concentrations of anti-EBNA-1 and anti-VCA IgG were measured by enzyme-linked immunosorbent assay (ELISA) using commercially available ELISA kits (NovagnostTM EBV-EBNA1 IgG and EBV-VCA IgG, cod. num. EBVG0580DB and EBVG0150DB, respectively) as published before [15]. Mocrotiter strip wells were precoated with recombinant EBNA-1 and synthetic VCA (p18) antigens, respectively. Briefly, a reference curve was generated in each assay using six serial dilutions of pooled high-positive serum samples ranging between 0.1 and 2.0 OD. CSF and corresponding serum prediluted with a range of 1:2 to 1:6 and 1:100 to 1:1200, respectively, were dispensed in duplicate into two microtiter plates, one precoated with highly purified EBV-EBNA-1 and the other precoated with EBV-VCA. A reference curve was generated in each assay using the standard serial dilutions by plotting the standard well concentrations, expressed as AU, versus the relative OD values. The lowest and the upper standard values of each plate were considered as 3.125 and 100 AU, respectively. The linear relationship between the AU values and the OD titers provides for easy extrapolation of concentration values. For each sample, anti-EBNA-1 and anti-VCA IgG concentrations were obtained by multiplying AU value for the corresponding dilution factor. Within-assay and between-assay precisions were determined after ten repeated measures into the same plate and by repetition of the same sample in ten consecutive plates, respectively. Both intra- and inter-assay variations, expressed as coefficient of variations (CV) %, were less than 8% for both anti-EBNA-1 and anti-VCA IgG.

### Calculation of Epstein-Barr nuclear antigen 1 and anti-viral capsid antigen IgG -specific antibody index

Local synthesis of anti-EBNA-1 and anti-VCA IgG was determined by an antibody index (AI) [30]. AI means the ratio between CSF and serum AU values (Q_Spec_) and CSF/serum total IgG levels expressed as mg/dl (Q_IgG_) in accordance to the following formula:

1 $$ \begin{array}{c}\mathrm{AI}={\mathrm{Q}}_{\mathrm{Spec}}/{\mathrm{Q}}_{\mathrm{IgG}}\hfill \\ {}\hfill \mathrm{with}\kern0.3em {\mathrm{Q}}_{\mathrm{Spec}}={\mathrm{AU}}_{\mathrm{CSF}}/{\mathrm{AU}}_{\mathrm{serum}}\ \mathrm{and}\ {\mathrm{Q}}_{\mathrm{IgG}}={\mathrm{IgG}}_{\mathrm{CSF}}/{\mathrm{IgG}}_{\mathrm{serum}}\hfill \end{array} $$

In the case of intrathecal synthesis of total IgG, QIgG appeared more elevated than Reiber’s hyperbolic discrimination line (Q_Lim_). Q_Lim_ represented the completely blood-derived CSF total IgG fraction calculated from the individual Q_Alb_ of a single patient. Therefore, owing to the introduction of this barrier-related correction of the Q_IgG_, the ratio was between Q_Spec_ and Q_Lim_ in agreement with the formula:

2 $$ \begin{array}{l}\begin{array}{c}\mathrm{AI}={\mathrm{Q}}_{\mathrm{Spec}}/{\mathrm{Q}}_{\mathrm{Lim}}\hfill \\ {}\hfill \mathrm{with}\ {\mathrm{Q}}_{\mathrm{Lim}}=0.93\;\sqrt{{\left({\mathrm{Q}}_{\mathrm{Alb}}\right)}^2+6{}^2{10}^{\hbox{-} 6}\;\hbox{--}\;1.7{}^2{10}^{\hbox{-} 3}}\hfill \end{array}\hfill \\ {}\hfill \end{array} $$

Therefore, to avoid false-negative results Equation 1 (Q_IgG_ < Q_Lim_) is used when no significant intrathecal total IgG synthesis occurs, while Equation 2 (Q_IgG_ > Q_Lim_) was preferred in case of intense brain-derived total IgG production in CSF. The EBV-specific intrathecal IgG synthesis was assumed for values of AI greater than 1.5.

### Antigen-specific immunoblotting

EBV-specific IgG OCB were investigated by antigen-specific immunoblotting (ASI) as reported elsewhere [31] using, as the target antigen, a crude viral lysate containing a high concentration of EBV antigens, including VCA, EBNA, early antigen diffuse (EA-D) and early antigen restricted (EA-R). Briefly, equal amounts of CSF and serum samples, at the same IgG concentrations, were applied to the agarose gel. After the IEF run, the gel was blotted onto a nitrocellulose sheet previously coated overnight at room temperature with EBV antigens (EBV antigen, cod. 11-511-248315-1, Genway, US) diluted in phosphate buffer (PBS) at the concentration of 100 μg/ml. The nitrocellulose membrane was then washed with distilled water and subsequently saturated by incubation in physiologic solution containing 2% bovine serum albumine (BSA) for 30 minutes at room temperature, in constant agitation. The antigen-specific blotting was then performed according to the manufacturer’s instructions. Nonspecific binding sites were further blocked in saline buffer containing 2% BSA for 30 minutes. The immunoblot was then incubated for 30 min at room temperature with peroxidase conjugated rabbit anti-human IgG diluted in 0.2% BSA saline. After another washing cycle with PBS, the blots were stained using peroxidase conjugated substrate of the kit according to the manufacturer’s instructions. The immunoblotting specificity was evaluated by testing CSF from a patient with subacute sclerosing panencephalitis that served as a control antigen to exclude nonspecific binding. No cross-reactivity to EBV was detected. As described for total OCB patterns, antigen-specific immunoblotting IgG banding patterns were categorized by two independent investigators (MC and EF).

### Determination of affinity distributions of Epstein-Barr virus-specific antibodies

CSF and serum affinity distributions of anti-EBNA-1 and anti-VCA IgG were measured at the same conditions by performing a ELISA protocol based on the employment of increasing concentrations of sodium thiocyanate (NaSCN) using the above-mentioned ELISA kits [32–34]. Briefly, 100 μl of each CSF or serum sample were added to four adjacent wells of each antigen-specific precoated ELISA microplate. After 1 h incubation at 37°C and three washing cycles, four consecutive dilutions of NaSCN (range: 0 M, 1.25 M, 2.5 M, and 5 M, where 0 M corresponded to 0.4% saline buffer) were applied into the wells corresponding to each patient for 10 min at 37°C. After incubation, the NaSCN was removed, and after three washing cycles wells were blocked by adding 100 μl per well of 2% BSA for 30 min at 37°C. After incubation, the BSA was discarded, and the detection antibody reaction, color development and absorbance recordings were performed as described in ELISA assay for serum and CSF levels of anti-EBNA-1 and anti-VCA IgG. Positive and negative controls and appropriate background wells (blanks) were included in each test. The average optical density between the blanks values was subtracted from all readings. The affinity distribution histograms for EBNA-1 and VCA-specific IgG were obtained assuming that, for each CSF and serum sample, the results observed in the first well, in which NaSCN were absent, represented the total antigen-specific IgG. In each series of 4 subsequent NaSCN dilutions per row, we divided the difference in OD values between two successive wells (for example, well 1 and well 2) by OD values of the total antigen-specific IgG, and multiplied by 100. In this way, the variation in OD values between two subsequent wells was expressed as the percentage of OD values of the total antigen-specific IgG measured in the first well. According to Luxton and coworkers [34], the difference in OD values between two consecutive wells reflected the proportion in which the bounded IgG molecules were reduced by the higher concentration of NaSCN dispensed in the two wells, and the corresponding percentage was considered as the relative affinity (RA) of the antibodies. When OD values measured in the well with higher levels of NaSCN were slightly greater compared to those detected in the adjacent well with lower levels of NaSCN, then it was assumed to be equivalent and the RA thus assumed to be zero. When positive OD values were still measurable in the wells with the highest concentration of NaSCN (5 M in the fourth well), we compared these OD values with those of a simulated following well, which was considered to be zero. Based on this principle, we obtained an RA value showing the proportion of antigen-specific IgG, which was not removed by the highest concentration (5 M) of NaSCN, and then the highest antibody affinity. On this principle, for each sample, we determined: RA1: percentage of IgG removed by 1.25 M NaSCN; RA2: percentage of IgG removed by 2.5 M NaSCN; RA3: percentage of IgG removed by 5 M NaSCN; RA4: percentage of IgG remaining after treatment with 5 M NaSCN. CSF and serum samples with RA value more than 2.5% from well number four containing the highest concentration (5 M) of NaSCN (RA4) were considered as having high-affinity anti-EBNA-1 and anti-VCA IgG, whereas CSF and serum samples with RA4 value equal or less than 2.5% were regarded as showing low-affinity anti-EBNA-1 and anti-VCA IgG. In addition, to increase the sensitivity in the detection of antibody binding affinity, the affinity of anti-EBNA-1 and anti-VCA IgG was also defined in all the samples by an affinity ratio (AR), a single numerical value, which relates the percentages of high affinity antibodies (RA4) to the percentages of low affinity antibodies (RA1):

$$ \mathrm{Affinity}\ \mathrm{R}\mathrm{atio}\;\left(\mathrm{A}\mathrm{R}\right)=\mathrm{R}\mathrm{A}4/\mathrm{R}\mathrm{A}1\times 100 $$

### Determination of Epstein-Barr virus-specific IgG oligoclonal band affinity

Affinity of EBV-specific IgG OCB was evaluated following a previously published immunoblotting protocol that employed increasing concentrations of NaSCN [35]. CSF samples were loaded in two parallel agarose gels (two groups of four samples per gel). IEF was performed for both gels at the same time and conditions in order to reduce errors due to interassay variation. After focusing, the gels were blotted onto the EBV precoated Ultrabind 450 membrane (Gelman Sciences Inc, Ann Arbor, MI, US) for 45 minutes under a 1 kg weight. Antigen precoating was performed overnight as illustrated in the ASI protocol. An UltraBind membrane was used due to its ability to bind covalently to antigens, which could be stripped by NaSCN from the nitrocellulose membrane, where their bind is usually non-covalent. Each membrane was then divided into two identical parts, each with four samples. Each part was incubated with four different concentrations of NaSCN (0 M, 1.25 M, 2.5 M, and 5 M, respectively) for 30 min at room temperature. The membranes were then washed in running tap water to remove any residual NaSCN and reincubated in blocking solution (BSA) for 30 minutes to ensure that any binding sites uncovered by the NaSCN incubation were blocked. Conjugated rabbit anti-human IgG diluted in 0.2% BSA saline was then added and after another washing cycle with PBS, the blots were stained using peroxidase-conjugated substrate (ASI protocol). Afterwards, the affinity of the EBV-specific IgG OCB was judged qualitatively based on the intensity of the bands detected by visual inspection. A persistence of EBV-specific IgG OCB at 5 M NaSCN was interpreted as being suggestive of high affinity.

### Statistics

Statistical analysis was performed with GraphPad Prism™. After checking data for normality by using the Kolmogorov- Smirnov test, a normality of data distribution was rejected in several variables. Therefore, statistical analysis was performed by a non-parametric approach. More precisely, continuous variables were compared using the Kruskal- Wallis test followed by the Mann- Whitney *U* test, whereas categorical variables were compared by means of a Chi-square test (*χ*2). Correlation between the continuous variables was assessed by the Spearman rank correlation coefficient test. Bonferroni correction was utilized for multiple comparisons. A value of *P* <0.05 was accepted as statistically significant.

## Results

### Cerebrospinal fluid and serum levels and intrathecal synthesis of anti-Epstein Barr virus IgG in relapsing-remitting multiple sclerosis patients and controls

Detectable CSF levels of anti-EBNA-1 IgG were statistically more frequent in RRMS than in NIND (*P* <0.05), whereas measurable CSF amounts of anti-VCA IgG were more frequent in OIND than in RRMS (*P* <0.001) without any significant differences among RRMS, OIND and NIND for serum quantifiable levels of anti-EBNA-1 and anti-VCA IgG (Table 3). As listed in Table 4, CSF levels of anti-EBNA-1 and anti-VCA IgG were statistically different among RRMS, OIND and NIND (Kruskal-Wallis: *P* <0.0001 and *P* <0.01, respectively). Significantly different levels of anti-EBNA-1 and anti-VCA IgG among RRMS, OIND and NIND were also observed in serum samples (Kruskal-Wallis: *P* <0.001 and *P* <0.05, respectively). Specifically, CSF anti-EBNA-1 IgG mean levels were more elevated in RRMS (*P* <0.01) and in OIND (*P* <0.05) than in NIND. CSF mean levels of anti-VCA IgG were higher in the OIND than in the RRMS and NIND groups (*P* <0.01 and *P* <0.0001, respectively). Serum anti-EBNA-1 IgG mean levels were more elevated in RRMS than in OIND and NIND (*P* <0.0001). Conversely, no significant differences were found for serum anti-VCA IgG mean levels among the groups examined. When RRMS patients were grouped according to clinical and MRI activity, no statistical differences were observed between the RRMS patients with and without clinical and MRI evidence of disease activity for CSF and serum mean concentrations of anti-EBNA-1 and anti-VCA IgG. An inverse correlation (Spearman: r = −0.336) was found between serum anti-EBNA-1 IgG levels and EDSS. We did not observe further definite relationships between disease severity and duration and CSF and serum concentrations of anti-EBNA-1 and anti-VCA IgG in RRMS patients (Table 5). An intrathecal synthesis of anti-EBNA-1 IgG and anti-VCA IgG, as indicated by AI values greater than 1.5, was present in a small percentage of RRMS patients and controls (RRMS = 6%, OIND = 7%, and NIND = 2% for EBNA-1-specific AI; RRMS = 2%, OIND = 5%, and NIND = 1% for VCA-specific AI), without any statistical differences among the groups.

**Table 3:** **Frequency of cerebrospinal fluid (CSF) and serum samples with detectable levels of anti-Epstein Barr nuclear antigen 1 (EBNA-1) and anti-viral capsid antigen (VCA) IgG in patients with relapsing-remitting multiple sclerosis (RRMS), other inflammatory neurological disorders (OIND) and noninflammatory neurological disorders (NIND)**

	**Anti-EBNA-1 IgG**		**Anti-VCA IgG**	
	**CSF**	**Serum**	**CSF**	**Serum**
RRMS (n = 100)	96/100 (96%)^a^	99/100 (99%)	65/100 (65%)	90/100 (90%)
OIND (n = 100)	91/100 (91%)	96/100 (96%)	86/100 (86%)^b^	94/100 (94%)
NIND (n = 100)	86/100 (86%)	94/100 (94%)	74/100 (74%)	90/100 (90%)

CSF anti-EBNA-1 IgG (Chi-square with Bonferroni correction): ^a^RRMS versus NIND (*P* <0.05); CSF anti-VCA IgG (Chi-square with Bonferroni correction): ^b^OIND versus RRMS (*P* <0.001).

**Table 4:** **Cerebrospinal fluid (CSF) and serum levels (median and IQR) and intrathecal synthesis of anti-Epstein Barr nuclear antigen 1 (EBNA-1) and anti-viral capsid antigen (VCA) IgG in patients with relapsing-remitting multiple sclerosis (RRMS), other inflammatory neurological disorders (OIND) and noninflammatory neurological disorders (NIND)**

	**Anti-EBNA-1 IgG**			**Anti-VCA IgG**		
	**CSF levels (AU)**	**Serum levels (AU)**	**AI >1.5**	**CSF levels (AU)**	**Serum levels (AU)**	**AI >1.5**
	**Median; IQR**	**Median; IQR**	**n/total (%)**	**Median; IQR**	**Median; IQR**	**n/total (%)**
Total RRMS (n = 100)	79.4; 37.5 to 174.3^a^	52,056; 23,274 to 94,815^c, d^	6/100 (6%)	36.4; 0.0 to 74.2	18962; 10659 to 46669	2/100 (2%)
CA RRMS (n = 77)	74.8; 38.2 to 167.7	41,783; 21,479 to 83,254	4/77 (5.2%)	41.9; 0.0 to 83.1	22546; 12433 to 51763	0/77 (0%)
CS RRMS (n = 23)	100.4; 51.0 to 272.7	59833; 36072 to 261044	2/23 (8.7%)	21.1; 0.0 to 59.9	14240; 6632 to 32515	2/23 (8.7%)
Gd + RRMS (n = 37)	90.4; 36.7 to 238.7	39325; 21479 to 105629	3/37 (8.1%)	23.7; 0.0 to 60.9	16836; 12476 to 37484	0/37 (0%)
Gd- RRMS (n = 63)	79.4; 43.3 to 139.0	53092; 27500 to 84551	3/63 (4.8%)	38.9; 0.0 to 85.1	21335; 10036 to 47672	2/63 (3.4%)
OIND (n = 100)	73.1; 31.0 to 151.6^b^	23503; 10386 to 44089	6/100 (6%)	82.7; 30.3 to 294.1^e, f^	29938; 8846 to 65475	5/100 (5%)
NIND (n = 100)	49.7; 15.9 to 118.9	24383; 7252 to 54754	2/100 (2%)	39.1; 0.0 to 130.8	28056; 12281 to 53124	1/100 (1%)

CSF anti-EBNA-1 IgG levels (Mann- Whitney with Bonferroni correction): ^a^MS versus NIND (p <0.01); ^b^OIND versus NIND (p <0.05). Serum anti-EBNA-1 IgG levels (Mann- Whitney with Bonferroni correction): ^c^MS versus OIND (p <0.0001); ^d^MS versus NIND (p <0.0001). CSF anti-VCA IgG levels (Mann- Whitney with Bonferroni correction): ^e^OIND versus MS (p <0.0001); ^f^OIND versus NIND (p <0.01). AI >1.5, antibody index abnormal values indicative of EBV-specific intrathecal synthesis; AU, arbitrary units; CA, clinically active (presence of relapse at entry); CS, clinically stable (absence of relapse at entry); Gd +, appearance of gadolium enhancing lesions on magnetic resonance imaging (MRI); Gd*-* **,** no evidence of gadolium enhancing on MRI; IQR, Interquartile Range; MS, multiple sclerosis; NIND, noninflammatory neurological diseases; OIND, other inflammatory neurological diseases.

**Table 5:** **Correlations between cerebrospinal fluid (CSF) and serum levels of anti-Epstein Barr nuclear antigen 1 (EBNA-1) and anti-viral capsid antigen (VCA) IgG and disease severity and duration in 100 patients with relapsing-remitting multiple sclerosis (RRMS)**

	**EDSS**	**Disease duration (months)**
CSF anti-EBNA-1 IgG levels (AU)	r = −0.1642	r = 0.1181
CSF anti-VCA IgG levels (AU)	r = 0.2294	r = 0.1184
Serum anti-EBNA-1 IgG levels (AU)	r = −0.336*	r = 0.0963
Serum anti-VCA IgG levels (AU)	r = 0.1901	r = 0.1443

*Spearman: *P* <0.001.

AU, arbitrary units; EDSS Expanded Disability Status Scale.

### Epstein Barr virus-specific IgG oligoclonal bands in relapsing-remitting multiple sclerosis patients

**Figure 1:**
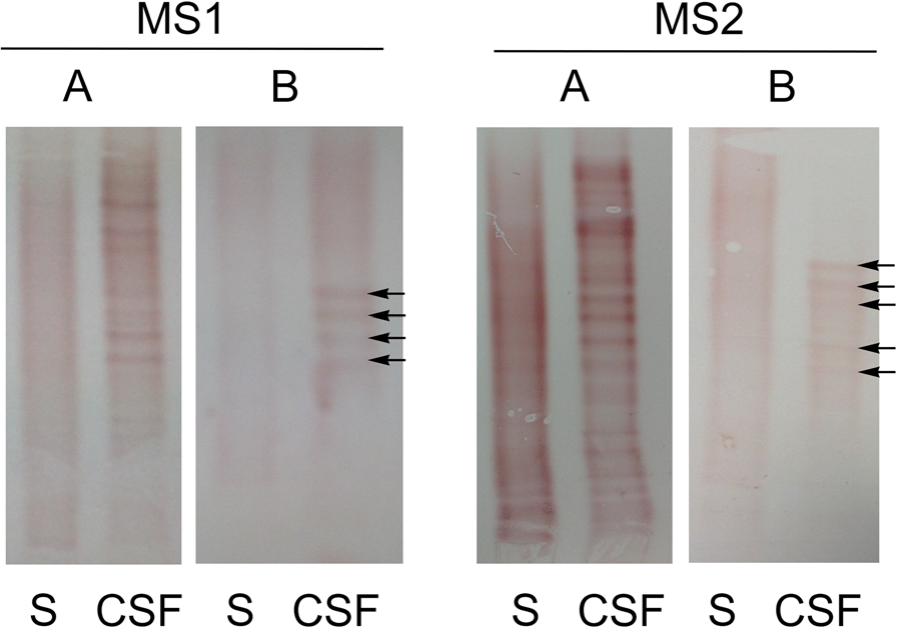
**Oligoclonal band profiles.** A comparison between total **(A)** and Epstein-Barr virus (EBV)-specific **(B)** IgG oligoclonal band (OCB) profiles obtained with isoelectric focusing (IEF) and antigen-specific immunoblotting, respectively, in paired cerebrospinal fluid (CSF) and serum samples of two relapsing-remitting multiple sclerosis (RRMS) patients. Corresponding bands are indicated by arrows. Among the RRMS patients with EBV-specific IgG OCB, CSF-restricted total IgG OCB were absent only in an RRMS patient with a ‘mirror’ pattern. In the remaining 24 RRMS patients with an EBV-specific intrathecal oligoclonal IgG synthesis, there was little overlap between total and virus-specific IgG OCB unique to CSF due to their high individual variability. More precisely, the EBV-specific OCB numbered fewer (mean ± SD = 3.6 ±1.9; range from 2 to 9) than total IgG OCB (mean ± SD = 9.9 ±4.6; range from 2 to 20), and did not always correspond to them because of the presence of additional bands in antigen-specific immunoblotting that were not visible in IEF.

EBV-specific IgG OCB were detected in 25/100 (25%) of the RRMS patients (Table 6). Four different profiles were recognized (normal; local synthesis; mixed and mirror). Among these, local synthesis and mixed patterns indicated an intrathecal synthesis of anti-EBV IgG OCB, whereas mirror pattern revealed an EBV-specific systemic oligoclonal response. Twenty-one RRMS patients had local synthesis, three had mixed and one had “mirror” patterns. Two illustrative cases describing paired total and EBV-specific IgG OCB from RRMS patients are reported in Figure 1. The number of bands and the ratio between EBV-specific and total IgG OCB showed great individual variability without any statistical correlations with clinical or MRI characteristics (data not shown). RRMS patients with and without intrathecally synthesized EBV-specific IgG OCB did not differ for disease duration and severity, clinical and MRI activity, and CSF and serum anti-EBNA-1 and anti-VCA IgG concentrations, with the exception of anti-VCA IgG concentrations in serum, which were greater in ASI negative than in ASI positive RRMS (Mann- Whitney: *P*<0.05). None of the patients with OIND and NIND showed OCB specifically directed against EBV, not even those with total IgG OCB (nine OIND and two NIND with local synthesis, one OIND and one NIND with mixed, eight OIND and seven NIND with mirror and three OIND with monoclonal patterns).

**Table 6:** **Demographic and clinical features, and cerebrospinal fluid (CSF) and serum levels and intrathecal synthesis of anti-Epstein Barr nuclear antigen 1 (EBNA-1) and anti-viral capsid antigen (VCA) IgG levels in 100 relapsing-remitting multiple sclerosis (RRMS) patients divided according to antigen-specific immunoblotting (ASI) findings**

	**ASI positive RRMS**	**ASI negative RRMS**
	**(n = 24)**	**(n = 76)**
Sex: F/M	17/7	53/23
Age, years: mean ± SD	36.7 ±9.1	37.5 ±11.1
Disease duration, months: mean ± SD	28.7 ±40.2	30.8 ±48.5
Disease severity, EDSS: mean ± SD	2.2 ±1.5	2.0 ±1.2
CA MS: n/total (%)	20/24 (83.3%)	57/76 (75.0%)
CA MS: n/total (%)	4/24 (16.7%)	19/76 (25.0%)
Gd+ MS: n/total (%)	13/24 (54.2%)	24/76 (31.6%)
Gd- MS: n/total (%)	11/24 (45.8%)	52/76 (68.4%)
CSF anti-EBNA-1 IgG (AU): mean ± SD	193.0 ±266.0	155.2 ±232.1
CSF anti-VCA IgG (AU): mean ± SD	41.6 ±56.9	81.5 ±243.4
Serum anti-EBNA-1 IgG (AU): mean ± SD	126502 ±205623	132114 ±198699
Serum anti-VCA IgG (AU): mean ± SD	21572 ±24497	50122 ±75151^a^
EBNA-1 AI >1.5: n/total (%)	3/21 (12.5%)	3/76 (4.0%)
VCA AI >1.5: n/total (%)	1/21 (4.2%)	1/76 (1.3%)

Serum anti-EBNA-1 IgG levels (Mann- Whitney): ^a^ASI negative versus ASI positive (p <0.05). AI >1.5, antibody index abnormal values suggestive of EBV-specific intrathecal synthesis; ASI negative, absence of EBV-specific IgG oligoclonal bands; ASI positive, presence of EBV-specific IgG oligoclonal bands; CA, clinically active (presence of relapse at entry); CS, clinically stable (absence of relapse at entry); EDSS, Expanded Disability Status Scale; Gd+, appearance of gadolium enhancing lesions on magnetic resonance imaging (MRI); Gd*-*, no evidence of gadolium enhancing on MRI; RRMS, relapsing-remitting multiple sclerosis; SD, standard deviation.

### Affinity distributions of anti-Epstein Barr nuclear antigen 1 and anti-viral capsid antigen IgG in relapsing-remitting multiple sclerosis patients and controls

**Figure 2:**
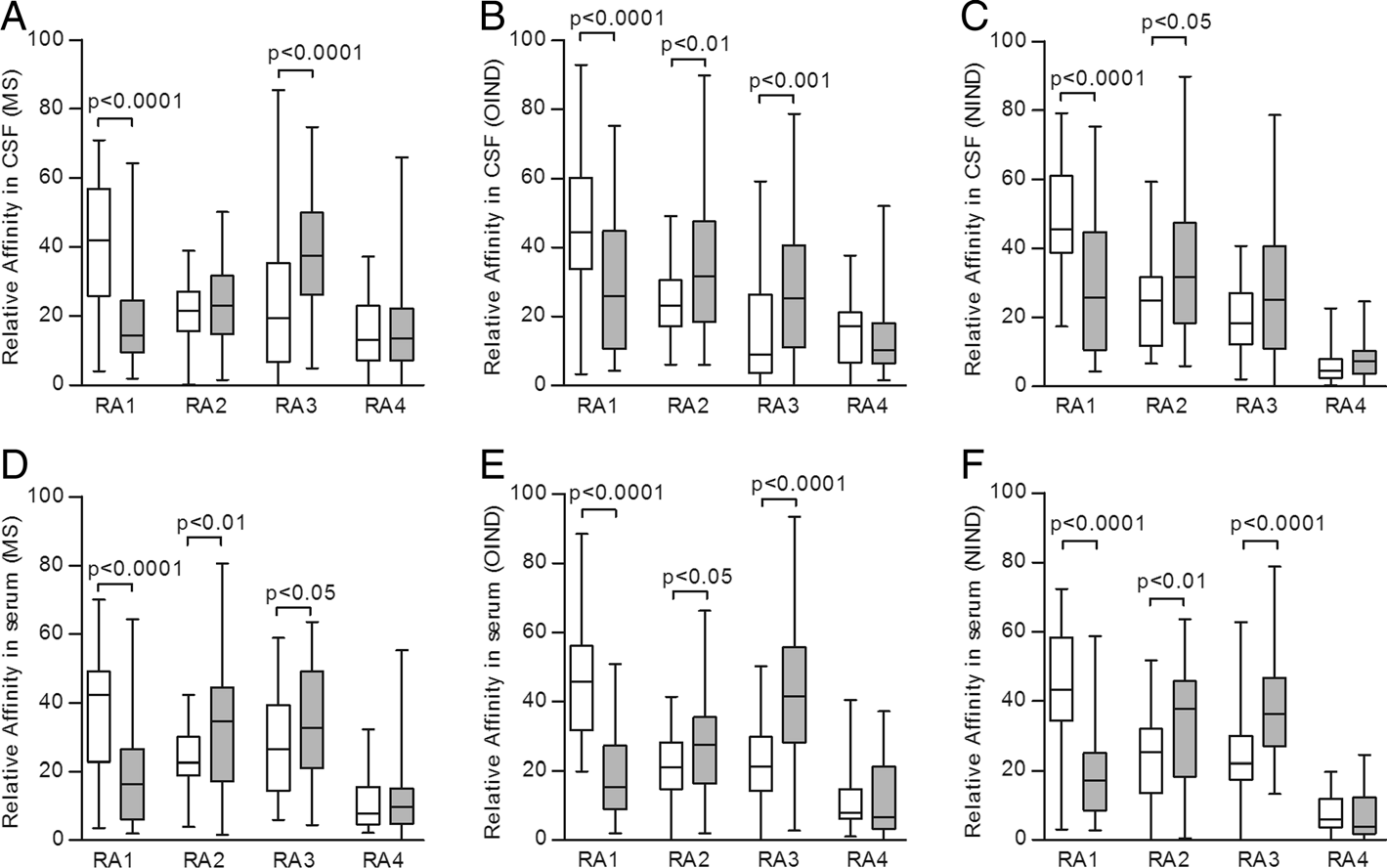
**Relative affinity distributions.** Relative affinity (RA) of anti-Epstein Barr nuclear antigen 1 (EBNA-1) IgG (white boxes) and of anti-viral capsid antigen (VCA) IgG (gray boxes) values obtained with increasing concentrations of sodium thiocyanate (NaSCN) in cerebrospinal fluid (CSF) and serum from 50 relapsing-remitting multiple sclerosis (RRMS), 50 other inflammatory neurological disease (OIND) and 50 noninflammatory neurological disease (NIND) patients. CSF and serum RA1 values were higher for anti-EBNA-1 IgG than for anti-VCA IgG in RRMS (panels **A** and **D**; Mann- Whitney; *P* <0.0001), OIND (panels **B** and **E**; Mann- Whitney; *P* <0.0001) and NIND (panels **C** and **F**; Mann- Whitney; *P* <0.0001). CSF RA2 values were more elevated for anti-VCA IgG than for anti-EBNA-1 IgG in OIND (panel **B**; Mann- Whitney; *P* <0.01) and NIND (panel **C**; Mann- Whitney; *P* <0.05). Serum RA2 values were greater for anti-VCA IgG than for anti-EBNA-1 IgG in RRMS (panel **D**; Mann- Whitney; *P* <0.01), OIND (panel **E**; Mann- Whitney; *P* <0.05) and NIND (panel **F**; Mann- Whitney; *P* <0.01). CSF RA3 values were higher for anti-VCA IgG than for anti-EBNA-1 IgG in RRMS (panel **A**; Mann- Whitney; *P* <0.001) and OIND (panel **B**; Mann- Whitney; *P* <0.001). Serum RA3 values were more increased for anti-VCA IgG than for anti-EBNA-1 IgG in RRMS (panel **D**; Mann- Whitney; *P* <0.05), OIND (panel **E**; Mann- Whitney; *P* <0.0001) and NIND (panel **F**; Mann- Whitney; *P* <0.001). The boundaries of the boxes represent the 25th to 75th quartile. The line within the box indicates the median. The vertical lines above and below the box correspond to the highest and lowest values, excluding outliers. RA, percentage of IgG removed by: 1.25 M NaSCN (RA1), 2.5 M NaSCN (RA2), 5 M NaSCN (RA3), and percentage of IgG remaining after treatment with 5 M NaSCN (RA4).

**Figure 3:**
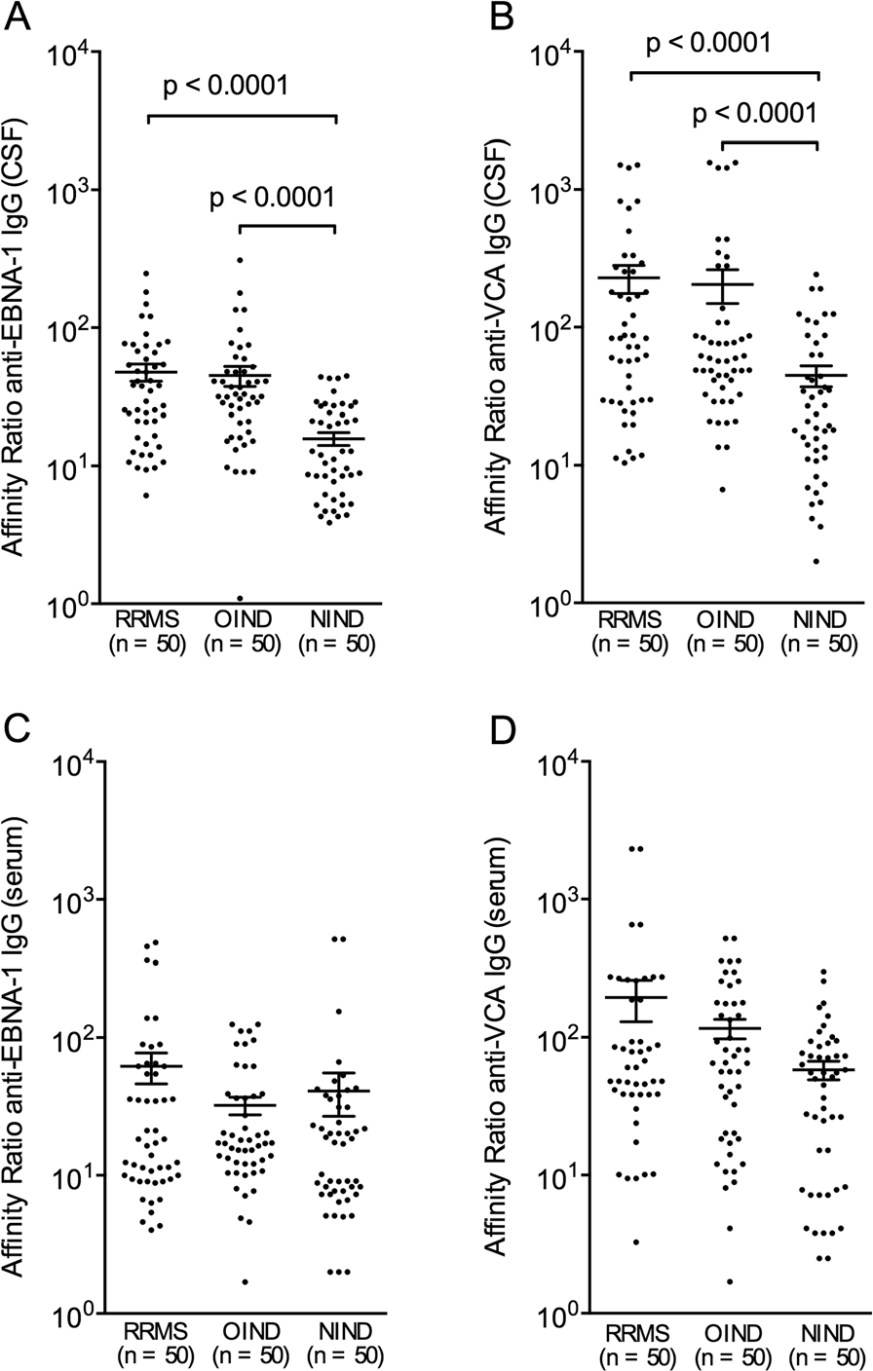
**Affinity ratio distributions.** Affinity ratio (AR) values of anti-Epstein Barr nuclear antigen 1 (EBNA-1) IgG and of anti-viral capsid antigen (VCA) IgG in cerebrospinal fluid (CSF) and serum from 50 relapsing-remitting multiple sclerosis (RRMS), 50 other inflammatory neurological disease (OIND) and 50 noninflammatory neurological disease (NIND) patients. Anti-EBNA-1 IgG and anti-VCA IgG CSF AR values were significantly higher in RRMS and OIND than in NIND (panels **A** and **B**; Mann- Whitney with Bonferroni correction; *P* <0.0001), without any further statistical differences between RRMS and controls for anti-EBNA-1 IgG and anti-VCA IgG serum AR values (panels **C** and **D**). Each point represents a single observation, excluding outliers. Horizontal bars indicate medians and error bars correspond to the standard error.

Affinity distributions of anti-EBNA-1 and anti-VCA IgG were quantitatively evaluated in the CSF and serum from a representative subpopulation of 50 RRMS (34 females and 16 males, mean age = 34.9 ± 8.2) and 50 OIND (34 females and 16 males, mean age = 35.5 ± 8.8), 50 NIND patients (34 females and 16 males, mean age = 36.1 ± 8.3) with high titers of both these EBV-specific antibodies. All RRMS, OIND and NIND patients with abnormal AI values and all RRMS patients with EBV-specific IgG OCB were included. CSF and serum relative affinity (RA) values obtained with increasing concentrations of NaSCN were significantly greater for anti-EBNA-1 IgG than for anti-VCA IgG at the lowest concentration of NaSCN while, on the contrary, for anti-VCA IgG than for anti-EBNA-1 IgG at higher NaSCN concentration. No differences were found at the highest concentration of NaSCN in all the three groups analyzed (Figure 2). As illustrated in Table 7, the CSF high-affinity anti-EBNA-1 IgG, as indicated by the RA4 value at 5 M NaSCN >2.5%, was significantly more frequent in RRMS (*P* <0.05) than in the NIND, whereas the CSF high-affinity anti-VCA IgG was statistically more represented in RRMS and OIND (*P* <0.05) than in NIND. On the other hand, serum high-affinity anti-VCA IgG was significantly more common in RRMS (*P* <0.01) than in NIND. No other statistical differences were found among the groups evaluated and between RRMS patients with and without clinical and MRI active disease. When we considered the affinity ratio (AR), the CSF for anti-EBNA-1 and anti-VCA IgG was statistically different among RRMS, OIND and NIND (Kruskal-Wallis: *P* <0.0001). More precisely, CSF AR values were significantly higher in RRMS and OIND than in NIND for anti-EBNA-1 and anti-VCA IgG (*P* <0.0001), without any further statistical differences between RRMS and controls for anti-EBNA-1 and anti-VCA IgG serum AR values (Figure 3).

**Table 7:** **Cerebrospinal fluid (CSF) and serum distributions of high-affinity anti-Epstein Barr nuclear antigen 1 (EBNA-1) and anti-viral capsid antigen (VCA) IgG in a subgroup of relapsing-remitting multiple sclerosis (RRMS), other inflammatory neurological disease (OIND) and noninflammatory neurological disease (NIND) patients**

	**RRMS**	**OIND**	**NIND**
	**(n = 50)**	**(n = 50)**	**(n = 50)**
CSF anti-EBNA-1 IgG: RA4 >2.5, n/total (%)	48/50 (96%)^a^	45/50 (90%)	40/50 (80%)
CSF anti-VCA IgG: RA4 >2.5, n/total (%)	48/50 (96%)^b^	47/50 (94%)^c^	40/50 (80%)
Serum anti-EBNA-1 IgG: RA4 >2.5, n/total (%)	47/50 (94%)	47/50 (94%)	48/50 (96%)
Serum anti-VCA IgG: RA4 >2.5, n/total (%)	44/50 (88%)^d^	40/50 (80%)	32/50 (64%)

CSF anti-EBNA-1, RA4 >2.5 (Chi-square with Bonferroni correction): ^a^RRMS versus NIND (*P* <0.05); CSF anti-VCA, RA4 >2.5 (Chi-square with Bonferroni correction): ^b^RRMS versus NIND (*P* <0.05) and ^c^OIND versus NIND (*P* <0.05); serum anti-VCA: RA4 >2.5 (Chi-square with Bonferroni correction): ^d^MS versus NIND (*P* <0.01). NIND noninflammatory neurological disorders; OIND, other inflammatory neurological disorders; RA4, percentage of IgG remaining after treatment with 5 M NaSCN; RRMS, relapsing-remitting multiple sclerosis.

### Affinity distributions of Epstein-Barr virus-specific oligoclonal antibodies

**Figure 4:**
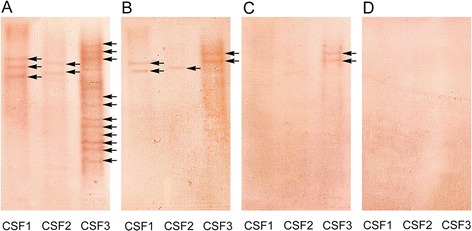
**Affinity distributions of Epstein-Barr virus (EBV)-specific IgG oligoclonal bands (OCB).** Changes in the intensity of EBV-specific IgG OCB (arrows) detected by antigen-mediated immunoblots (AMI) specific for EBV performed with the employment of different concentration of NaSCN. **A)** Untreated: 0 M NaSCN (saline buffer); **B)** 1.25 M NaSCN; **C)** 2.5 M NaSCN; **D)** 5 M NaSCN.

Affinity distributions of the EBV-specific IgG OCB were qualitatively assessed in CSF from all 24 RRMS patients with EBV-specific CSF-restricted oligoclonal IgG response. In all the CSF samples analyzed, EBV-specific IgG OCB were found to have low affinity since they completely disappeared after treatment with the highest concentration (5 M) of NaSCN. Particularly, in only 4 CSF samples EBV-specific IgG OCB persisted at a concentration of 2.5 M NaSCN, whereas in the remaining 20 CSF specimens EBV-specific IgG OCB were lost after treatment with 1.25 M NaSCN concentration. Figure 4 depicts three explicative cases showing the effects on intensity of EBV-specific OCB IgG of increasing concentrations of NaSCN in RRMS patients.

## Discussion

In this study, we investigated different aspects of CSF and serum EBV-specific humoral immune response in RRMS focusing on intrathecal production and affinity distributions of EBV-specific IgG. We here showed that concentrations of anti-EBNA-1 IgG were higher in RRMS and OIND than in NIND in CSF and in RRMS than in OIND and NIND in serum. Interestingly, an inverse correlation between serum levels of anti-EBNA-1 IgG and EDSS was observed in RRMS. In contrast, the CSF anti-VCA IgG levels were higher in OIND than in RRMS and NIND. No relationships were found between anti-EBNA-1 and anti-VCA IgG and clinical and MRI MS activity, and an intrathecal synthesis of anti-EBNA-1 and anti-VCA IgG were observed in a minority of RRMS patients and controls. These results confirm that, while serum levels of anti-EBNA-1 IgG can be considered as a hallmark of MS [5,6], elevated CSF amounts of anti-EBNA-1 IgG do not seem to be selectively associated with MS, but are shared by several inflammatory neurological conditions [15]. In accordance with our recent publication [15], but in contrast with other previous studies [8,9], our results do not support the value of serum titers of anti-EBNA-1 IgG as a biomarker for MS disease activity and progression but indicate that an increase in disability may be linked to a decline in systemic humoral reaction to EBNA-1. In addition, our findings further pointed out the poor significance of CSF and serum levels of anti-VCA IgG, although serum concentrations of anti-VCA IgG were repeatedly related to MS [5,6] and an association between them and MRI cortical atrophy was recently described [10]. On the other hand, the low rate of detection for an intrathecal release of anti-EBNA-1 and anti-VCA IgG documented in RRMS and controls was concordant with all [14–19] but two [13,20] prior investigations, providing additional evidence that EBV-specific intrathecally produced antibodies are innocent bystanders, reflecting a polyspecific humoral reactivity directed against many different pathogens not related to the cause of the disease and promoted by the overactive chronic immune stimulation associated with MS brain inflammation [14,19]. The divergences that emerged with previous studies [8–10,13,20] may derive from differences in patient selection and determination techniques, highlighting, moreover, the need for standardized protocols. The methodological issues could also explain the discrepancies found between our and some earlier investigations for CSF-restricted EBV-specific OCB that were recognized in MS patients [20–24]. We exclusively detected EBV-specific OCB restricted to CSF in 24% of RRMS patients, suggesting that, as qualitative analysis is more sensitive than quantitative in the determination of an intrathecal IgG synthesis [36], an EBV targeted intrathecal humoral immune response, which implies that an EBV persistent chronic brain infection may occur in a subset MS patients. However, our affinity qualitative studies demonstrated that CSF-restricted EBV-specific OCB had low affinity in all RRMS patients. These findings indicate that EBV-specific intrathecal oligoclonal high-affinity antibodies are absent in MS. Therefore, as the process of immune maturation promoted by an infection leads to a gradual increase in antibody affinity resulting in a strong production of high-affinity antibodies specifically directed against the causative pathogen [32], our observations collectively argue against the possibility that an EBV persistent brain chronic infection may exist in MS. This suggests that the widely accepted association between anti-EBNA-1 IgG and MS may not always correspond to causation [37]. Conversely, quantitative analysis based on RA4 value at 5 M NaSCN >2.5% and AR collectively demonstrated that CSF high-affinity anti-EBNA-1 and anti-VCA IgG were more frequent in RRMS and OIND than in NIND and were equally represented in RRMS and OIND. In the absence of an oligoclonal intrathecal production of high affinity antibodies, these results suggest the presence of an EBV-specific polyclonal humoral immune response operating in the CSF compartment that is not limited to MS, but represents a common feature of inflammatory CNS disorders, which is predominantly blood-derived, and, thus, likely reflects an antecedent EBV infection during puberty [38]. In addition, this reaction may be in part induced by a polyclonal activation of memory B cells and long-lived plasma cells that predominate within the inflammatory brain microenvironment [39]. Intriguingly, we observed, in both RRMS and controls, the occurrence of higher affinity anti-EBV viral surface protein (VCA) antibodies in comparison with those specific for nuclear viral proteins released from dying cells (EBNA-1). These results prove that EBV acts as an intermittently cytopathic virus [38]. In summary, this study has shown that, at intrathecal level, MS is generally marked by polyclonal high-affinity anti-EBV antibodies that passively transudate from serum. In a subset of patients (24%), there are additional EBV-specific oligoclonal low-affinity antibodies that are locally synthesized within the CNS. These data do not justify the hypothesis that EBV persistence leading to a productive infection in the brain can play a role in MS. Nevertheless, the presumed EBV persistent CNS infection may occur in a latent form that, unlike the chronic form, is characterized by an intermittent and not a continual antigen stimulation without a significant production of specific CSF and serum antibodies [40]. Thus, the possibility that EBV is a cofactor in the development and maintenance of the disease, sustaining the polyspecific intrathecal antibody synthesis typical of MS [41], cannot be completely excluded. In addition, in this study we did not explore other potential mechanisms that can trigger MS autoimmunity such as molecular mimicry consisting of a cross-reaction between EBV and CNS self-antigens [11], bystander damage due to a dysregulation of EBV infection within the brain [22] and accumulation of EBV infected autoreactive B cells in the CNS related to a decrease in systemic CD8 T cell control of EBV infection [3]. This study may be affected by other potential limitations. First, the lack of spinal cord MRI examinations suggests the possibility that some active lesions could be missed in our series, leading to a potentially inappropriate allocation of patients to the MRI inactive group. Second, the inability to quantify CSF anti-EBNA-1 and anti-VCA antibody fraction could not allow us to exactly discriminate between targeted and polyspecific intrathecal synthesis of microbial antibodies [19], leading to possible misinterpretations of increasing ASI values. Third, we did not evaluate ASI and CSF affinity distributions of specific-IgG for other infectious agents, such as measles, rubella, and varicella zoster, which are frequently detected in CSF of MS patients. Fourth, the affinity distribution of EBV-specific IgG was not semiqualitatively evaluated [33] and should be confirmed with BIAcore analysis, a technique that is more specific to describe affinity of antigen-specific IgG since it allows the determination of Kd values [42]. Thus, further studies are warranted for a better understanding of the role of EBV in MS pathogenesis.

## Abbreviations

AI: antibody index
AR: affinity ratio
ASI: antigen-specific immunoblotting
AU: arbitrary units
B-CSF-B: blood-cerebrospinal fluid barrier
BSA: bovine serum albumin
CD8+ T cell: cytotoxic T cell
CIS: clinical isolated syndrome
CNS: central nervous system
CSF: cerebrospinal fluid
DPTA: diethylenetriaminepentacetate
EA-D: early antigen diffuse
EA-R: early antigen restricted
EBNA-1: Epstein-Barr nuclear antigen 1
EBV: Epstein-Barr virus
EDSS: Expanded Disability Status Scale
ELISA: enzyme linked immunosorbent assay
Gd: gadolinium
IEF: isoelectric focusing
IgG: immunoglobulin G
IM: infectious mononucleosis
MRI: magnetic resonance imaging
MS: multiple sclerosis
NaSCN: sodium thiocyanate
NIND: noninflammatory neurological diseases
OCB: oligoclonal bands
OD: optical density
OIND: other inflammatory neurological diseases
PBS: phosphate buffer
QAlb: cerebrospinal fluid/serum albumin quotient
RA: relative affinity
RRMS: relapsing-remitting multiple sclerosis
VCA: viral capsid antigen
